# Voice Navigation Created by VIP Improves Spatial Performance in People with Impaired Vision

**DOI:** 10.3390/ijerph19074138

**Published:** 2022-03-31

**Authors:** Yu-Hsiu Hung, Kai-Yu Tsai, Eva Chang, Rain Chen

**Affiliations:** 1 Department of Industrial Design, National Cheng Kung University, Tainan City 701, Taiwan; idhfhung@mail.ncku.edu.tw (Y.-H.H.); kaiyu097@gmail.com (K.-Y.T.); 2 Program in Interdisciplinary Studies, National Sun Yat-sen University, Kaohsiung 804, Taiwan; evachang@mail.nsysu.edu.tw; 3 Department of Visual Communication Design, Southern Taiwan University of Science and Technology, Tainan City 710, Taiwan

**Keywords:** impaired people, landmarks, voice navigation

## Abstract

The difficulty associated with spatial navigation is one of the main obstacles to independent living for visually impaired people. With a lack of visual feedback, visually impaired people must identify information from the external environment through other sense organs. This study employed an observational survey to assess voice navigation version A, created by visually impaired people, and voice navigation version B, created by non-visually impaired people. Thirty-two simulated visually impaired people were assigned to conduct task assessments of voice navigation version A and version B. For mission 1, the mean completion rate is 0.988 ± 0.049 (version A); the mean error rate is 0.125 ± 0.182 (version A). For mission 2, the mean completion rate is 0.953 ± 0.148 (version A); the mean error rate is 0.094 ± 0.198 (version A). The assessment results concluded that version A has a higher completion rate (*p* = 0.001) and a lower error rate (*p* = 0.001). In the assessment of subjective satisfaction, all the indicators regarding the impression of navigation directives in version A were significantly superior to those indicators in version B. It appears that version A has a different logic of framing than version B. In future applications, a voice navigation version shall be built, according to the way visually impaired people think, because it will facilitate the direction guide when there is a lack of visual feedback.

## 1. Introduction

Golledge proved that the difficulty associated with spatial navigation is one of the main obstacles to independent living for visually impaired people [1]. Some authors have shown that visually impaired people (VIP) often need to explore indoors and outdoors, and to reach their destination, VIP must pass through some indoor or outdoor environments, or make route planning decisions [2]. VIP cannot precisely perceive objects in an environment or the objects’ relative positions, because they lack the concept of space.

Research has revealed that when people explore the environment, the layout of the space affects their exploration experiences [3]. When people seek roads in a complicated environment, they attempt to comprehend the overall planning and layout of the environment, and correspond the environmental information they perceive to the map of their mental imagery. Hence, having overall environmental information as a reference is crucial for road seekers. Without visual feedback, the mental map that VIP form is fragmentary after they collect environmental information. It has been observed that VIP cannot perceive spatial representation entirely and precisely, so they cannot form a complete cognitive map, which makes VIP find orientation and mobility difficult [4].

Chi found that, under the circumstance of having no visual feedback, VIP must perceive the external environment through other sensory perception, such as hearing, touch, taste, and smell. Many people think that once VIP lose their vision, their other sensory perception, such as hearing and touch, may be enhanced spontaneously [5]. However, VIP must spend a lot of time training to maximize their sensory perception, other than the sense of sight. The training includes how to pass through/by barriers in an environment and move to the destination safely.

Environmental information helps VIP determine directions and generate their mental imagery of the environment. When VIP are incapable of determining their walking paths with vision, their mental maps become an important basis for route planning. Environmental information can not only assist VIP in understanding, recognizing, and having control of the environment, but also makes it safer and more effective when VIP walk alone.

More and more evidence has shown that by integrating different sensory inputs (for example, hearing plus touch), the spatial memory representation structure generated herewith is highly similar to that formed by vision. The same results can be found in the performance of spatial tasks, which is called functional equivalence. After the spatial information was learned through vision and touch, the reference object was displaced for the task participants to point out the spatial positions [6]. The position of the target object changed after the spatial information was learned through spatial audio frequency or spatial language [7,8]. After the spatial information was learned through vision, spatial audio frequency, and spatial language, the target object was moved to a different position [9]. A study of neuroscience has demonstrated that VIP use the same brain area as sighted people when learning spatial information. VIP learn it through a tactile map, while sighted people learn it through a visual map [10].

In some research, voice information has been proven to be more useful than non-voice information in navigation. Neuville and Trassoudaine reported that words are more understandable to VIP, compared with directional non-verbal audio signals [11]. A study of the Talking Images project indicated that 66% of VIP do not think that a tactile map is helpful. The Royal National Institute of the Blind claimed that verbal directives are considered to be more helpful than a tactile map [12]. Golledge et al. noticed that VIP prefer being guided by voice information [13].

When stimulation from non-visual senses, such as touch, hearing, and smell, can convey the same spatial information as the visual sense, why do VIP still have trouble with orientation and mobility? The possible reason for this may be the amount of information conveyed by non-visual information or by visual information. Loomis et al. asserted that the perceptual range of vision is 500 times wider than that of touch [14]. From the viewpoint of the navigation process, vision can instantly capture a complete picture of an entire space and quickly determine the spatial relations during a person’s movement. However, VIP can only use non-visual senses, such as touch, hearing, and smell, to explore the environment. The distances that these non-visual senses can explore are relatively short, and the information perceived is vague and inaccurate.

In sum, VIP need to spend more time on spatial exploration. If voice navigation can help VIP have foreknowledge of the conditions in an environment, it may greatly reduce a VIP’s exploration time. Nowadays, VIP often use GPS navigation (such as Google maps and Apple maps) on their smart phones as a spatial reference when they walk. However, the voice information of the GPS navigation gives directions from the viewpoint of sighted people. This study asked two groups, visually impaired people (VIP) and non-visually impaired people (NVIP), to record two versions of voice navigation (version A and version B, respectively). Through experiments, verify the differences between the version A and the version B.

## 2. Method

Some authors have indicated that each individual’s spatial recognition relies on various factors, including personal characteristics (such as age, gender, and cognitive ability), environmental characteristics (such as extent, structure, and familiarity), and learning process (such as the way in which they acquire information, learning conditions, and information media) [15,16]. From the user’s point of view, the spatial guidance for people with normal vision may not be suitable for blind people. It is a very important issue to prove that the spatial guidelines established by blind people are more suitable for blind people to use. This study aimed to investigate the difference in voice navigation between the VIP’s version (visually impaired people, VIP) and NVIP’s version (non-visually impaired people, NVIP), so environmental characteristics and the learning process were controlled during the experiment. The details of the experiment follow.

### 2.1. Participants

The experiment had the following two phases: phase 1: VIP and NVIP established their own voice navigation versions; phase 2: sighted people executed designated missions for both voice navigation versions with their eyes covered [17].

In phase 1, participants were divided into the following two groups: visually impaired people (VIP) and non-visually impaired people (NVIP). In the group of VIP, there were four participants (coded as A1–A4), who have been blind for 7 years on average (vision = 0), with an average age of 37 years. In the group of NVIP, there were four participants (coded as B1–B4) with normal sightedness (vision ≥ 1), with an average age of 32 years.

In phase 2, 32 normal-sighted people, with an average age of 32 years (vision ≥ 1), were recruited to execute designated missions with their eyes blindfolded. The purpose was to simulate how normal-sighted people (simulated visually impaired people, SVIP) execute missions designed for voice navigation version A and version B, without visual feedback.

### 2.2. Experimental Design

**Figure 1:**
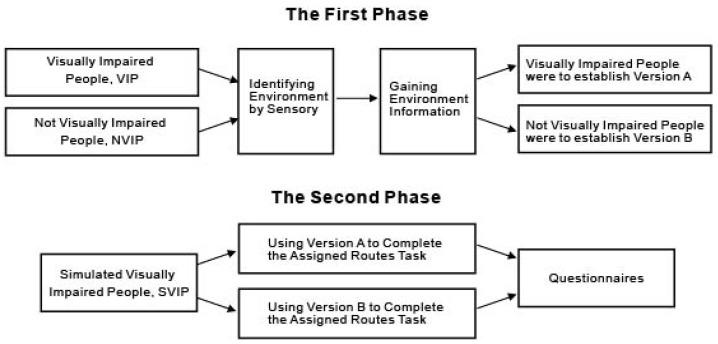
Procedures of the experiment.

There were two phases in the experiment. The aim of the first phase was to establish two voice navigation versions (VIP established version A; NVIP established version B). The purpose of version A is to explore the route according to the situation of VIP itself (VIP is blind, vision = 0). The purpose of version B is to explore the route according to the situation of NVIP itself (NVIP is normal-sighted people, vision ≥ 1). Both groups were allowed to freely explore the environment in their familiar ways, without a time limit, before they recorded voice directions for the navigation. The second phase was the SVIP’s execution of missions designed for voice navigation version A and version B. Firstly, SVIP listened to version A and version B randomly, and executed designated missions. SVIP then answered a subjective questionnaire for the researcher to analyze and find out which version satisfied SVIP more, version A or version B? Procedures of the experiment are shown in Figure 1.

### 2.3. Navigation Task

**Figure 2:**
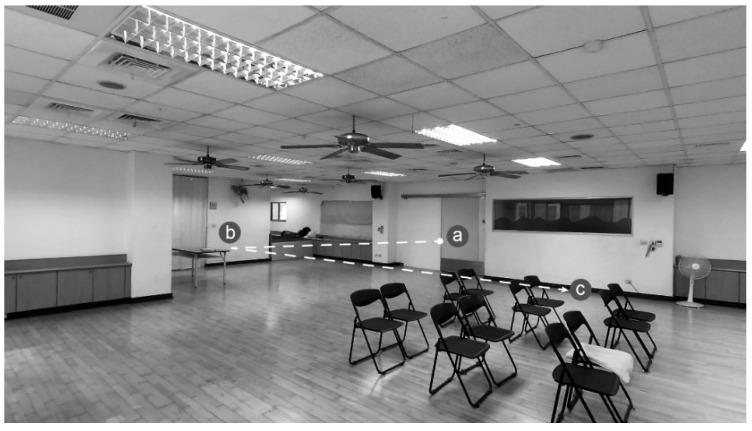
Exploration route of the spatial task.

In the study, VIP and NVIP were required to explore the task environment in advance. Their tasks were to record voice directions for the navigation. The navigation was used to guide SVIP from point a to point b (mission 1), and then to point c (mission 2). The route of exploration is shown in Figure 2.

### 2.4. Statistical Method

In this study, independent samples *t*-test was used to test the completion rate, error rate, and subjective satisfaction of version A and version B.

## 3. Results

### 3.1. Comparison of Version A and Version B

**Figure 3:**
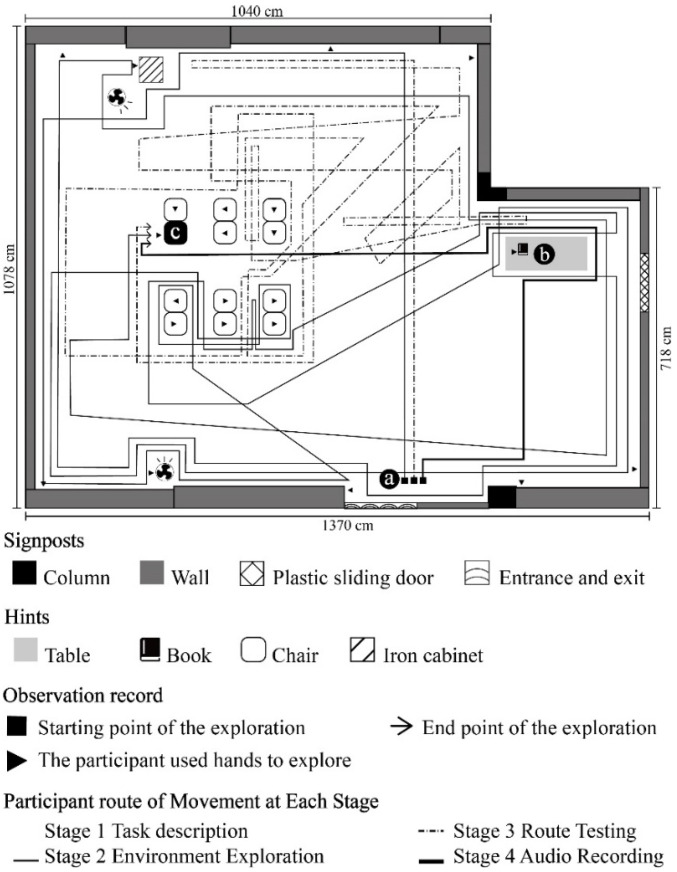
A1 participant’s route of movement at each stage.

**Figure 4:**
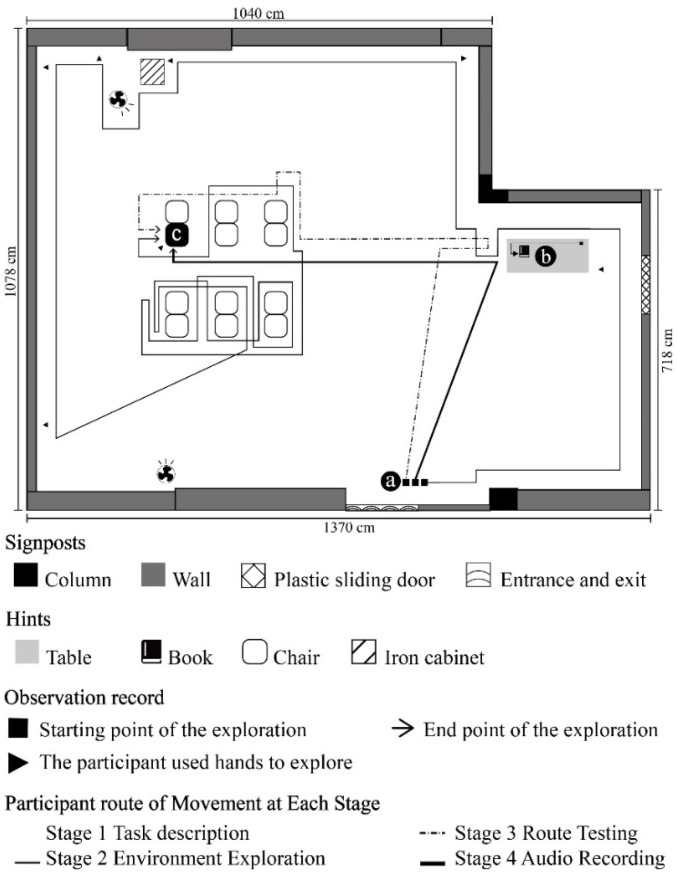
A2 participant’s route of movement at each stage.

**Figure 5:**
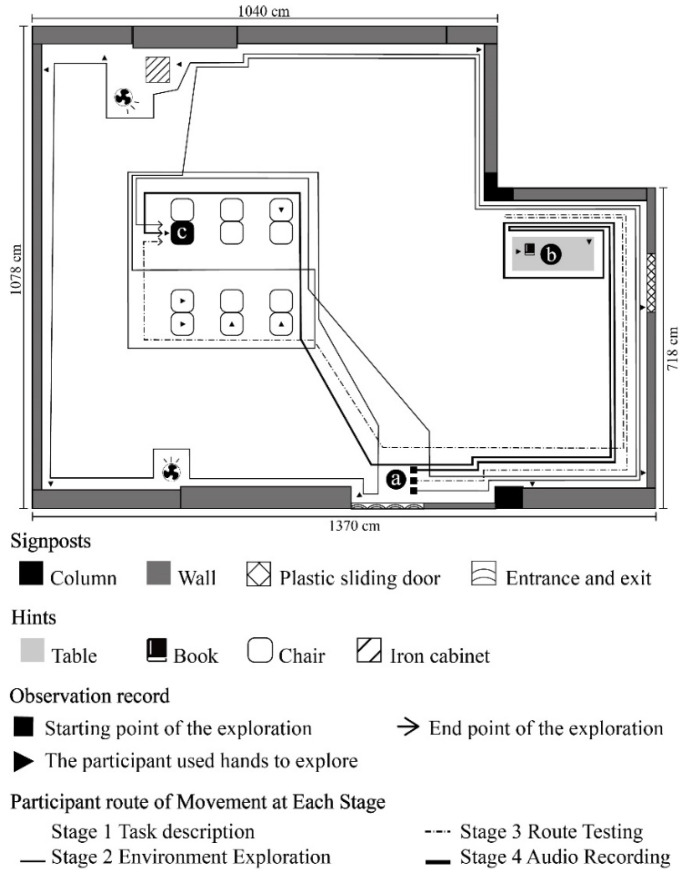
A3 participant’s route of movement at each stage.

**Figure 6:**
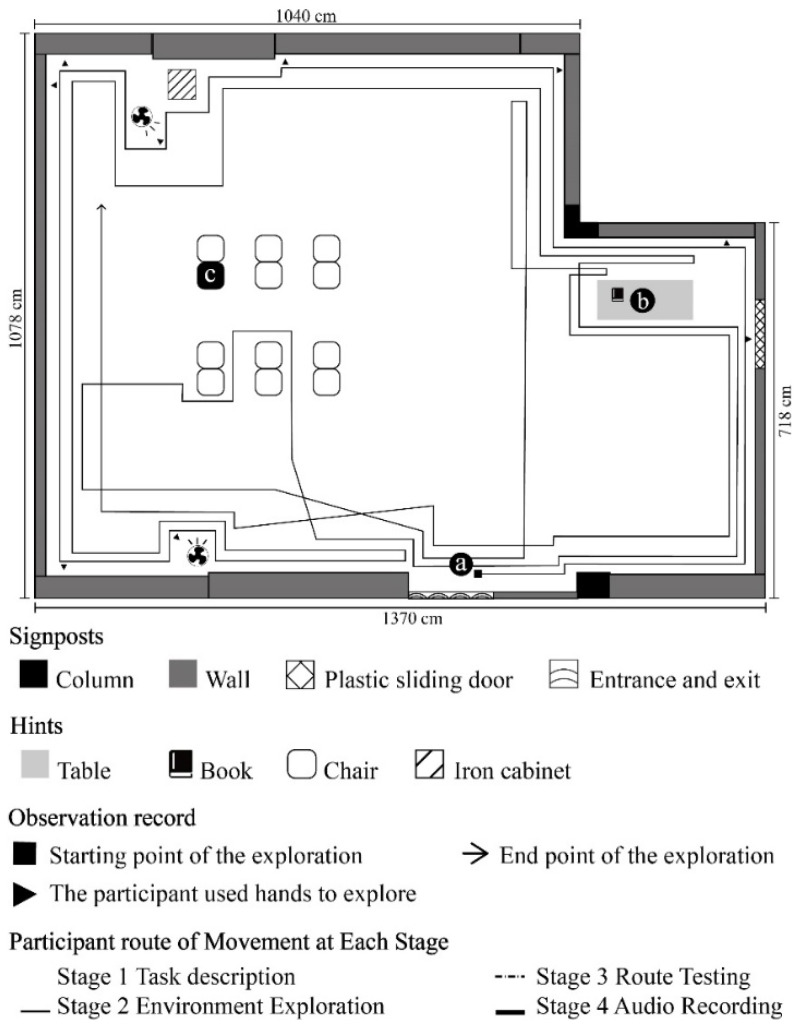
A4 participant’s route of movement at each stage.

**Figure 7:**
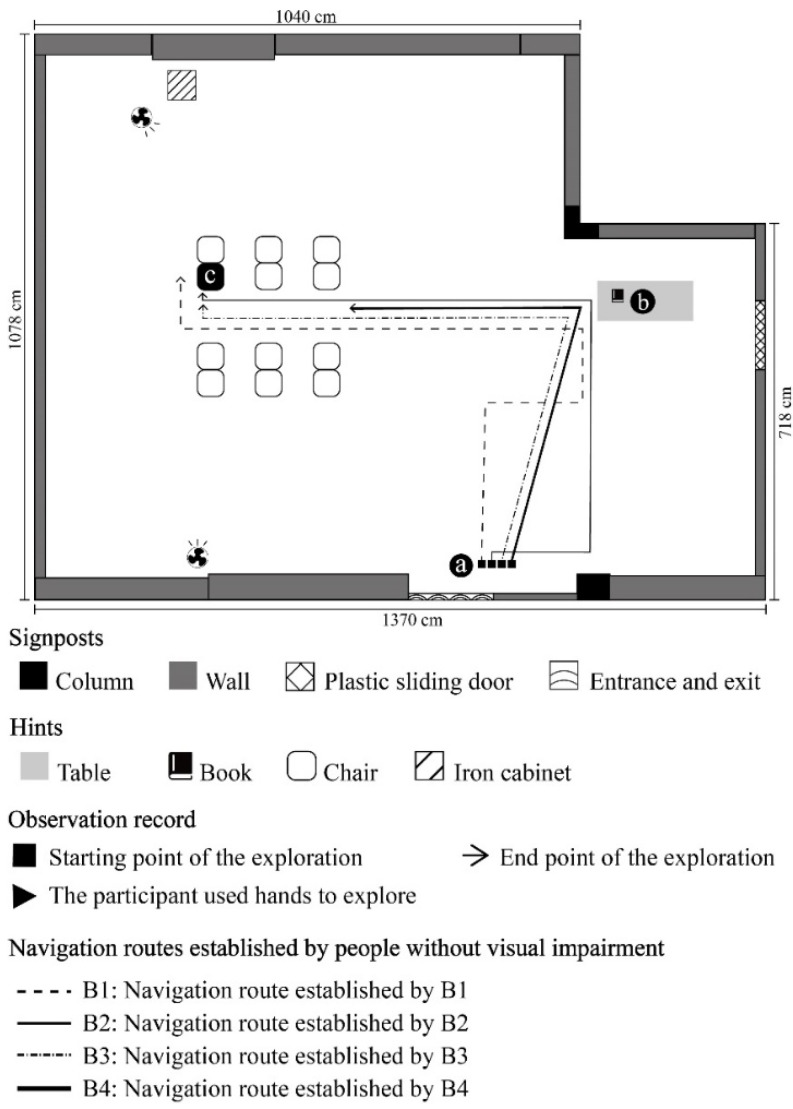
B1–B4 participant’s route of movement at each stage.

Mission 1: VIP/ NVIP to walk from point a to point b; Mission 2: VIP/ NVIP to walk from point b to point c. Figure 3, Figure 4, Figure 5 and Figure 6 show the exploration route of A1–A4, respectively. Figure 7 shows the exploration route of B1–B4, respectively. In the group of NVIP, their routes of exploration were more consistent, because they all had normal sight.

As a summary of the two voice navigation versions, VIP tended to tap the environment with their canes and slowly formed the environmental information, and NVIP utilized their visual determination to directly and rapidly understand the environment. In voice navigation version A, it was obvious that VIP gave more directions about landmarks. The content manifested that VIP highly depend on information about landmarks for environmental positioning. In voice navigation version B, the directions that NVIP gave were mostly based on self-orientation; for example, they were accustomed to counting the number of their paces. Table 1 is a comparison between version A and version B.

### 3.2. Paired Samples t-Test between Version A and Version B

This study used the paired samples t-test to compare play times, error times, completion time, and mission completion rate between version A and version B (as shown in Table 2).

The assessment of mission 1 showed that (1) the play times of version B were greater than the play times of version A (*p* = 0.042); (2) the navigation error times of version B were greater than those of version A (*p* = 0.042); (3) the completion time of version A was longer than that of version B (*p* < 0.001); (4) the completion rate of version A was higher than that of version B (*p* = 0.036). To sum up, version A was significantly superior to version B in most assessments, except the completion time. The results suggested that voice navigation version A, recorded by VIP, was more acceptable to SVIP, although it took them more time to complete the mission.

The assessment of mission 2 showed that (1) the play times of version B were greater than the play times of version A (*p* < 0.001); (2) the navigation error times of version B were greater than those of version A (*p* < 0.001); (3) the completion time of version A was longer than that of version B (*p* = 0.068); (4) the completion rate of version A was higher than that of version B (*p* < 0.001). To sum up, version A was significantly superior to version B in most assessments, except the completion time. The results suggested that voice navigation version A, recorded by VIP, was more acceptable to SVIP, although it took them more time to complete the mission.

### 3.3. Subjective Satisfaction of Version A and Version B

Concerning the impression of navigation directives (as shown in Table 3), the measurement results indicated that (1) when using version A for navigation, SVIP felt a better sense of security than when they used version B (*p* = 0.002); (2) when using version A for navigation, SVIP felt a better sense of direction than when they used version B (*p* = 0.001); (3) when using version A for navigation, SVIP felt a better level of clarity than when they used version B (*p* < 0.001); (4) when using version A for navigation, SVIP felt a better level of effectiveness than when they used version B (*p* < 0.001). To sum up, version A was significantly superior to version B in these four assessments. The results suggested that voice navigation version A, recorded by VIP, was more acceptable to SVIP, concerning the impression of navigation directives.

Regarding the understandability of navigation directives (as shown in Table 4), the measurement results indicated that (1) when using version A for navigation, the SVIP’s comprehension of mission 1 was worse than when they used version B (*p* = 0.325), but the difference was not significant; (2) when using version A for navigation, the SVIP’s comprehension of mission 2 was worse than when they used version B (*p* = 0.385), but the difference was not significant; (3) the SVIP’s satisfaction was higher in general when using version A than when using version B (*p* = 0.083), but the difference was not significant. To sum up, there was no significant difference in the understandability of navigation directives between version A and version B.

The measurement results concluded that version A was significantly superior to version B in the impression of navigation directives; there was no significant difference in the understandability of navigation directives between version A and version B.

## 4. Discussion

The reason that VIP have mobility difficulties results not from physical impairment, but from being unable to collect a large amount of environmental information, which makes the identification of conditions in the environment and smooth mobility difficult for VIP. VIP, whether their impairment is congenital or acquired, must learn and verse themselves in the way of non-vision-based mobility. Blind people, owing to them having no vision at all, must rely on non-visual senses to explore the environment and determine their directions when they walk. Consequently, voice navigation becomes one of the most important assistants to VIP.

This study employed an observational survey to investigate how VIP and NVIP explore the environment and then create their own version of voice navigation (version A was created by visually impaired people; version B was created by non-visually impaired people).

Allen explained that landmarks can help people orient and keep themselves on the right path in a certain direction. Some researchers have discovered that VIP’s way of memorizing landmarks is different from NVIP [18]. VIP tend to choose landmarks that have more sensory cues as rememberable points. For example, when VIP enter an indoor environment from outdoors, the change in temperature or in sunlight can provide VIP with useful cues to determine directions. However, when VIP stay indoors, there are fewer cues that can help determine directions (temperature and light in a room are usually steady), landmarks that VIP can use become not clear, and changes in each landmark are hard to perceive.

Most NVIP do not introspect during navigation. NVIP usually rely on visual perception to move freely in an environment. When they move, they also use other senses to explore the environment. Most of the time, they still rely on visual information (such as road signs, traffic lights, and road conditions) to determine directions and make decisions. Vision has been considered to be crucial in seeking road, especially in orientation [19]. Plikynas et al. presented an approach to enhance electronic traveling aids for people who are blind or severely visually impaired, using indoor orientation [20]. Relevant studies have shown that blind people rely on Bluetooth technology [21], wave radar [22], and so on when navigating in space. The integration of technology has become very important [23]. The results of this study can be applied in similar fields.

## 5. Conclusions

In this study, SVIP assessed the difference in voice navigation between the VIP’s version A and NVIP’s version B. In the results of task performances, the number of audio words in version A was 2.4 times greater than that in version B. For SVIP, the mission completion time for version A was longer than for version B. With respect to other quantitative indicators, especially the completion rate, version A was significantly superior to version B. In the measurement results of subjective satisfaction, SVIP considered that version A delivers a better sense of security and direction, and a higher level of clarity and effectiveness. Nevertheless, there was no significant difference in the understandability of navigation directives between version A and version B. This study concluded that the version of voice navigation created by VIP is more suitable for SVIP. In future applications, a voice navigation version shall be built, according to the way VIP think, because it will facilitate the direction guide when they lack visual feedback.

## Figures and Tables

**Table 1:** A comparison between version A and version B.

Description	VIP (Version A)	NVIP (Version B)
Distance	It was marked by the change in different landmarks.	It was described by counting the number of paces.
Direction	Adjustments in directions were made based on the hints of landmarks.	Directions were given based on self-orientation.
The number of audio words	202 words.	84 words.
In sum	Positions of landmarks in the environment were the standards of orientation.	Self-orientation and task points were the standards of orientation.

**Table 2:** Execution results of mission 1 and mission 2.

Items	Version	Mission 1 (a to b)				Mission 2 (b to c)			
		Mean	SD ^1^	*T* Value	*p* Value	Mean	SD ^1^	*T* Value	*p* Value
Play Times	A	1.132	0.181	−2.12	0.042	1.281	0.309	−10.72	<0.001
	B	1.297	0.356	--	--	1.938	0.246	--	--
Error Times	A	0.125	0.182	−2.13	0.042	0.094	0.198	−16.12	<0.001
	B	0.281	0.335	--	--	0.938	0.246	--	--
Completion Time	A	75.079	17.240	15.09	<0.001	78.875	56.985	1.89	0.068
	B	33.688	11.779	--	--	59.125	29.001	--	--
Completion Rate	A	0.988	0.049	2.19	0.036	0.953	0.148	5.67	<0.001
	B	0.906	0.198	--	--	0.469	0.507	--	--

^1^ Standard deviation.

**Table 3:** The impression of navigation directives.

Items	Version	Mean	SD ^1^	*T* Value	*p* Value
The Sense of Security	A	4.219	0.975	3.478	0.002
	B	3.219	0.906	--	--
The Sense of Direction	A	4.188	0.931	3.754	0.001
	B	3.250	0.950	--	--
The Level of Clarity	A	4.031	0.999	4.487	<0.001
	B	2.719	1.054	--	--
The Level of Effectiveness	A	4.219	0.751	4.693	<0.001
	B	3.063	1.045	--	--

^1^ Standard deviation.

**Table 4:** The understandability of navigation directives.

Items	Version	Mean	SD ^1^	*T* Value	*p* Value
Comprehension of Mission 1	A	4.625	0.554	−1.000	0.325
	B	4.750	0.568	--	--
Comprehension of Mission 2	A	3.906	1.058	−0.882	0.385
	B	4.094	0.995	--	--
General Satisfaction	A	4.188	0.780	1.791	0.083
	B	3.813	1.230	--	--

^1^ Standard deviation.

## Data Availability

Data can be requested from the corresponding author.
